# Effect of Body Position on Dynamic Apparent Diffusion Coefficient Changes During the Cardiac Cycle in the Human Brain

**DOI:** 10.1002/jmri.29758

**Published:** 2025-03-21

**Authors:** Naoki Ohno, Tosiaki Miyati, Masatomo Uehara, Riho Okamoto, Mitsuhito Mase, Satoshi Kobayashi

**Affiliations:** ^1^ Faculty of Health Sciences, Institute of Medical, Pharmaceutical and Health Sciences Kanazawa University Kanazawa Japan; ^2^ Department of Radiological Technology Okinawa Miyako Hospital Miyakojima Japan; ^3^ Radiology Division Kanazawa University Hospital Kanazawa Japan; ^4^ Department of Neurosurgery Nagoya City University Graduate School of Medical Sciences Nagoya Japan; ^5^ Department of Radiology Kanazawa University Hospital Kanazawa Japan

**Keywords:** apparent diffusion coefficient, diffusion‐weighted imaging, multi‐posture MRI

## Abstract

**Background:**

Dynamic changes in the apparent diffusion coefficient (ΔADC) during the cardiac cycle reflect water molecule fluctuations in the brain and intracranial conditions. While body position strongly affects intracranial conditions, the relationship between ΔADC and body position has been less explored, as conventional MRI is typically performed only in the supine position.

**Purpose:**

To investigate ΔADC and mean ADC (ADC_mean_) of the brain in supine and sitting positions using a multi‐posture MRI system.

**Study Type:**

Prospective.

**Subjects:**

Nine healthy volunteers (all males; mean age, 23.5 years).

**Field Strength/Sequence:**

0.4 T, electrocardiographically synchronized single‐shot diffusion echo‐planar imaging sequence with *b*‐values of 0 and 500 s/mm^2^.

**Assessment:**

ADC maps were generated at multiple cardiac phases in each subject in the sitting and supine positions. For each position, an ADC_mean_ map was then generated as the voxel‐wise mean ADC across all phases, and a ΔADC map was generated as the voxel‐wise maximum difference in ADC across phases. ΔADC and ADC_mean_ were measured in 2 frontal and 2 occipital lobe regions and averaged. ΔADC, ADC_mean_, and heart rate (HR) were compared between supine and sitting positions.

**Statistical Tests:**

Wilcoxon signed‐rank test. Significance was set at *p* < 0.05.

**Results:**

Both ΔADC and ADC_mean_ were significantly higher in the sitting position compared with the supine position (ΔADC: 0.84 ± 0.06 × 10^−3^ mm^2^/s vs. 0.68 ± 0.05 × 10^−3^ mm^2^/s; ADC_mean_: 0.87 ± 0.02 × 10^−3^ mm^2^/s vs. 0.79 ± 0.06 × 10^−3^ mm^2^/s, respectively). These increases were consistent across all participants. In addition, HR was significantly higher in the sitting position compared with the supine position (73.8 ± 8.4 bpm vs. 58.1 ± 3.7 bpm).

**Data Conclusion:**

ΔADC and ADC_mean_ of the brain are significantly higher in the sitting position than in the supine position.

**Evidence Level:** 2.

**Technical Efficacy:** Stage 1.

1

**Plain Language Summary**

- How body position changes affect the brain is not fully understood.
- This study investigated how body position affects water movement in the brain using a special MRI scanner that works in various body positions.
- We measured dynamic apparent diffusion coefficient changes (ΔADC) during the cardiac cycle, which reflects water molecule fluctuation, in nine healthy young men in supine and sitting positions.
- We found that ΔADC was significantly higher when sitting compared to supine. This suggests that gravity influences how water molecules fluctuate in brain tissue.
- This finding is relevant for understanding the effects of body position on the brain.

## 2 Introduction

The interplay between cerebral blood flow (CBF), intracranial pressure (ICP), compliance, and water diffusion—the hemodynamic and biomechanical properties of the brain—is important for maintaining cerebral homeostasis [1, 2, 3]. Accurate monitoring of these properties is essential for effective patient care and intervention [4]. MRI is a powerful tool for the noninvasive assessment of functional information of the brain, for example, CBF, ICP, and water diffusion [4, 5]. These interconnected parameters offer a comprehensive evaluation of the hemodynamic and biomechanical properties of the brain [6, 7, 8, 9]. Among these, water diffusion is particularly important as it provides unique insights into the microstructural environment of brain tissue, reflecting changes in cellular density, membrane integrity, and interstitial space [5]. Thus, measuring diffusion contributes to a comprehensive evaluation of the brain's physiological state.

Diffusion‐weighted imaging (DWI) is an important MRI technique for understanding water mobility within tissues, which is particularly useful for early stroke detection and brain tumor diagnosis [10, 11]. However, its sensitivity to macroscopic movements presents challenges in accurately capturing microscopic water diffusion in the brain. Bulk motion (pulsatile brain motion synchronized with the cardiac cycle) can introduce artificial phase dispersion in DWI, leading to an overestimation of the apparent diffusion coefficient (ADC) [12, 13]. To mitigate this issue, Brockstedt et al. demonstrated the effectiveness of single‐shot echo‐planar imaging (EPI) in minimizing the impact of bulk motion on ADC measurements and showed stable ADC values in the brain across different cardiac phases [14]. Conversely, Nakamura et al. observed significant ADC changes in the white matter during the cardiac cycle, synchronizing with intracranial volume change (ICVC), despite combining single‐shot EPI with parallel imaging, rectangular field of view, and half‐scan methods, which further reduce motion sensitivity by shortening the data sampling window [15].

ICVC during the cardiac cycle is a dynamic process driven by the interplay of three key components entering and leaving the cranium: arterial blood flow, venous outflow, and cerebrospinal fluid (CSF) flow [4]. ADC changes during the cardiac cycle can be attributed to the following mechanisms [16, 17]: each heartbeat introduces arterial inflow into the cranium, causing a transient increase in intracranial volume loading. To compensate for this volume loading, venous blood and CSF outflow from the cranium occur, inducing brain pulsation. Concurrently, water molecules in the brain are impacted and fluctuate due to the loading force propagated into the brain parenchyma, resulting in dynamic ADC changes (ΔADC) during the cardiac cycle.

A recent study by Sloots et al. has provided further evidence that ΔADC likely reflects physiological processes rather than brain motion artifacts [18]. Notably, elevated ΔADC has been observed in patients with idiopathic normal‐pressure hydrocephalus (iNPH), indicative of reduced intracranial compliance, compared with age‐matched healthy controls and patients with asymptomatic ventricular enlargement [16]. These findings indicate the potential of ΔADC as a noninvasive marker for assessing the biomechanical properties of the brain.

While hemodynamic and biomechanical properties have been assessed in the supine position, body position can affect these measurements. Differences in CBF, CSF dynamics, and ICP have been observed between supine and sitting positions, highlighting the differential impact of gravity on the human body in these positions [19, 20, 21]. For instance, transitioning from a supine to a sitting position has been shown to decrease ICP due to hydrostatic pressure changes within the craniospinal axis [22].

Given the sensitivity of ΔADC to the biomechanical properties of the brain, it is likely that body position similarly influences this parameter. However, the relationship between ΔADC and body position is unclear, as conventional MRI scans are typically performed only in the supine position.

Thus, the aim of this study was to investigate potential differences in ΔADC between supine and sitting positions using a multi‐posture MRI system capable of scanning in various body positions [23].

## 3 Materials and Methods

### 3.1 Participants

The institutional review board approved this study (approval no. 561–4) and all participants provided written informed consent prior to MRI scans.

**FIGURE 1:**
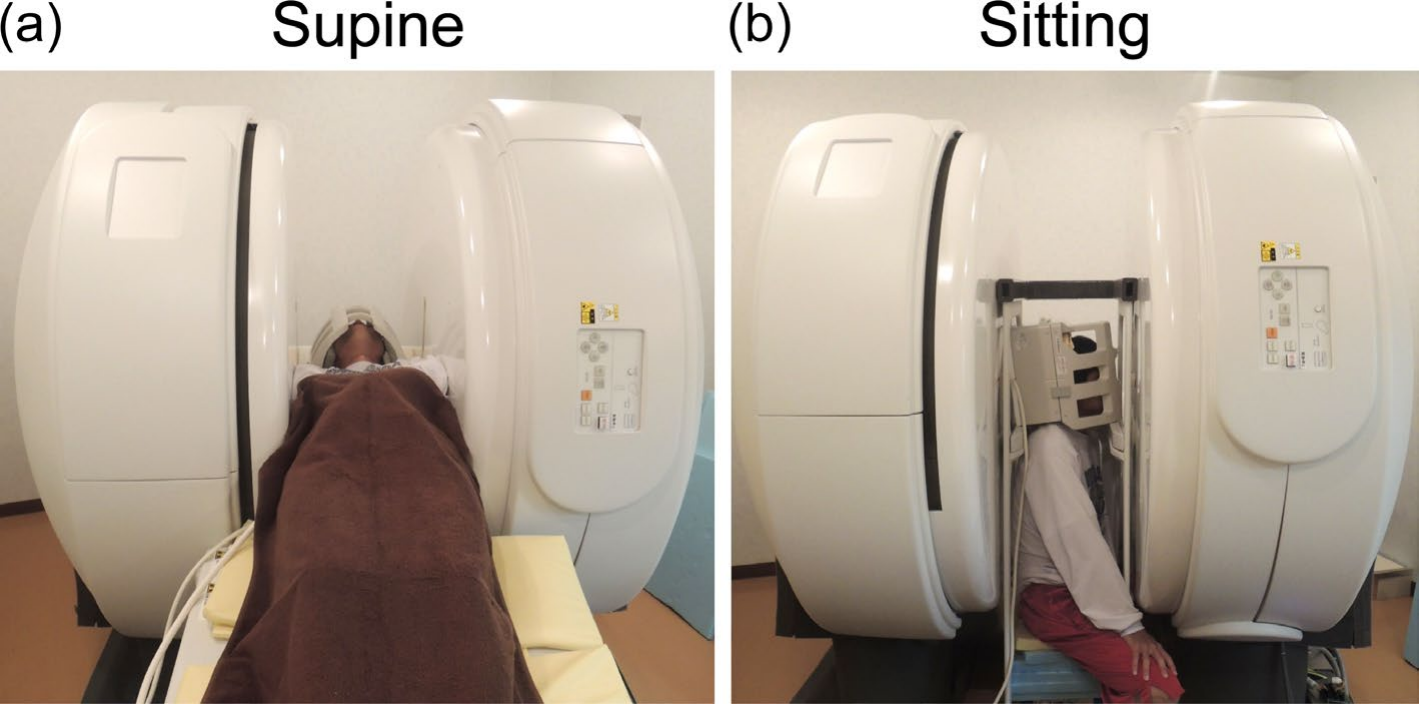
(a) Supine and (b) sitting positions in multi‐posture MRI.

Nine healthy males (average age, 23.5 years; range 21–28 years) were enrolled in this study. Inclusion criteria were ages 20–30 years and no history of neurological disorders or head trauma. Participants underwent MRI scans in two body positions: supine and sitting in a fixed order (Figure 1). Between scans, they were asked to move away from the scanner for at least 10 min to allow for physiological adaptation.

### 3.2 MRI Acquisition

Using a 0.4 T multi‐posture MRI system with a quadrature head coil (FUJIFILM Healthcare, Tokyo, Japan), transverse diffusion‐weighted images at the basal ganglion level were obtained in different cardiac phases. Head motion was minimized by using foam and inflatable pads between the subject's head and the receiver coil. Cardiac synchronization was achieved using electrocardiogram (ECG) triggering, with the R‐wave serving as the trigger. The following imaging parameters were chosen to minimize bulk motion: pulse sequence, ECG‐synchronized single‐shot diffusion EPI; repetition time, two R‐R intervals; echo time, 114.1 ms; imaging matrix, 64 × 64; field of view, 256 mm × 256 mm; slice thickness, 4 mm; flip angle, 90°; number of signals averaged, 2; b‐values, 0 and 500 s/mm^2^. Diffusion gradients were applied in three orthogonal directions (x, y, and z axes). Additionally, a half‐scan technique was implemented with a factor of 0.6 to reduce the bulk motion effect by shortening the data sampling window. To reduce signal saturation and allow for recovery of longitudinal magnetization, the TR was set to two R‐R intervals. Consequently, data acquisition was performed only during the first R‐R interval, with no data acquisition during the second R‐R interval. Moreover, only one cardiac phase was acquired per TR, and multiple acquisitions with different trigger delays were performed to sample different cardiac phases. The trigger delay was set at regular intervals (30 ms), resulting in 22–37 acquisitions per subject, depending on heart rate (HR).

### 3.3 Analytical Procedures

**FIGURE 2:**
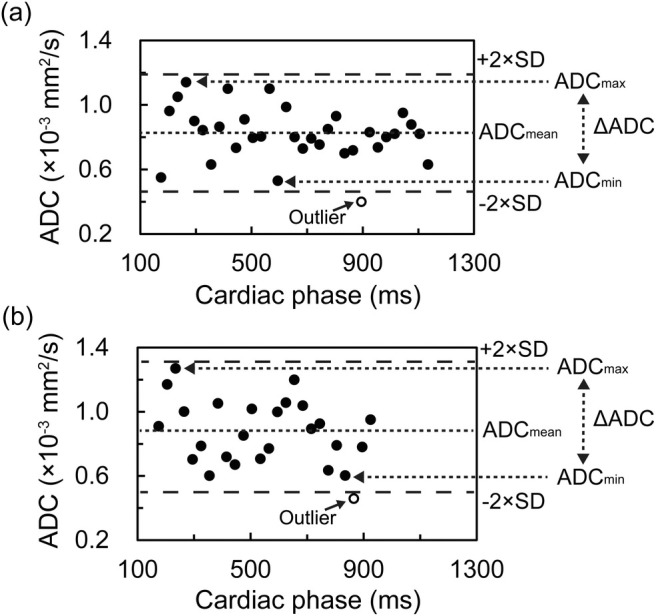
ADC changes during the cardiac cycle in (a) supine and (b) sitting positions, calculated from mean ADC values within regions of interest. Data are shown for a representative subject.

For each cardiac phase, an ADC map was calculated using the following equation:

(1) $$\mathrm{ADC}=\ln\left(S_{1}/S_{2}\right)/500$$

where *S*_1_ and *S*_2_ represent signal intensities of DWI at b‐values of 0 and 500 s/mm^2^, respectively. Figure 2 shows representative ADC changes over the cardiac cycle in one subject for both supine and sitting positions, calculated from mean ADC values within regions of interest (ROIs) that are described later in this section. Then, a ΔADC map was created by calculating the voxel‐wise difference between the maximum ADC (ADC_max_) and minimum ADC (ADC_min_) across all cardiac phases using the following equation:

(2) $$∆\mathrm{ADC}={\mathrm{ADC}}_{\max}-{\mathrm{ADC}}_{\min}$$

The selection of ADC_max_ and ADC_min_ values was based solely on the highest and lowest ADC values observed across all cardiac phases, regardless of the specific phase of the cardiac cycle; however, outliers exceeding 2 standard deviations from the mean were excluded from this selection to improve the reliability of ΔADC measurements. Furthermore, a mean ADC (ADC_mean_) map was generated by calculating the mean ADC value across all cardiac phases.

**FIGURE 3:**
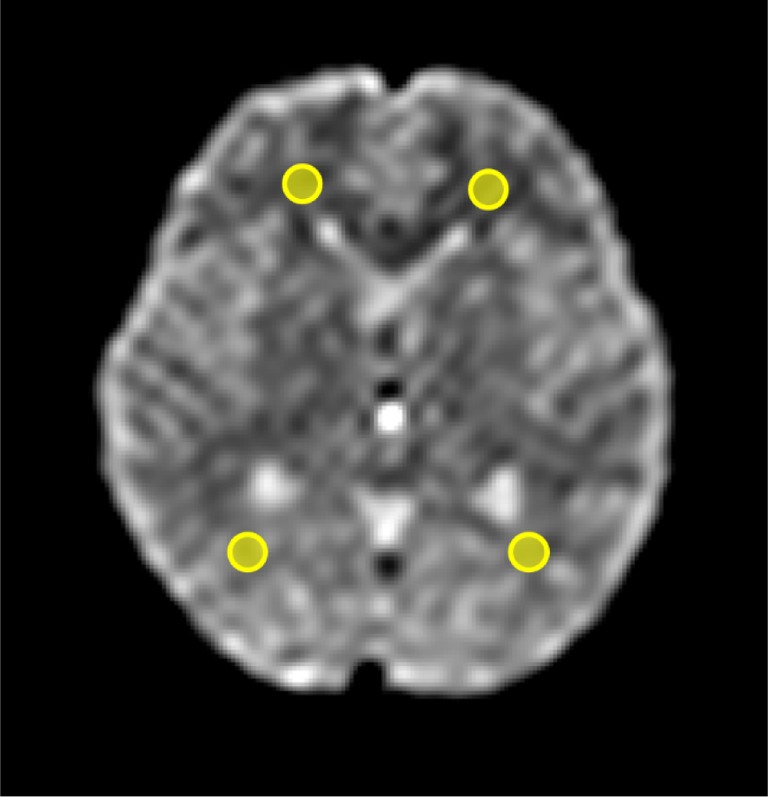
Regions of interest (yellow circles) in the frontal and occipital lobes on the b0 image.

For each subject, ROIs were placed in the frontal and occipital white matter directly on the ΔADC and ADC_mean_ maps. These regions were selected for their relatively homogeneous white matter structures, minimizing partial volume effects, and because previous studies have reported cardiac cycle‐dependent ADC changes in white matter [8, 15, 16, 17, 18]. ROI placement was guided by reference to a corresponding b0 image at the same slice level to ensure accurate localization and to exclude CSF and other structures (Figure 3). This resulted in four ROIs per subject, and the mean ΔADC and ADC_mean_ values across these four ROIs were calculated to obtain a single representative value for each subject in each body position. The ROI size was standardized at 52 mm^2^ to facilitate consistent analysis and simplify comparisons. To determine measurement reliability, two independent observers (M.U. and N.O., with 3 and 17 years of experience in neuro MRI, respectively) independently placed the ROIs. The ROIs were consistently placed in the same anatomical locations for both the supine and sitting positions. The mean ΔADC and ADC_mean_ within the ROIs, as well as the mean HR during DWI scans, were compared between the supine and sitting positions.

To confirm sufficient data fidelity and evaluate the potential impact of motion during image acquisition, signal‐to‐noise ratio (SNR) measurements were also performed using the same ROIs as those used for the ΔADC and ADC_mean_ analyses. The SNR was calculated as the quotient of the mean signal intensity across the four ROIs and the mean standard deviation of the background noise, multiplied by a correction factor of 0.66 to account for the Rayleigh distribution. The background noise was determined by placing four ROIs of the same size (316 mm^2^) at the four corners of the image, outside the brain, in areas free from signal and artifacts.

### 3.4 Statistical Analysis

To assess the consistency between the two observers' measurements, intraclass correlation coefficients (ICCs) were calculated for ΔADC and ADC_mean_ values in both the supine and sitting positions using a two‐way mixed effects model with absolute agreement. The ICCs were interpreted as follows: > 0.90, excellent; 0.75–0.9, good; 0.5–0.75, moderate; and < 0.5, poor consistency. The Wilcoxon signed‐rank test was performed to evaluate posture‐related changes in ΔADC, ADC_mean_, and HR, with statistical significance set at *p* < 0.05. These statistical analyses were performed using IBM SPSS Statistics (version 25, IBM, Armonk, NY, USA). Additionally, a post hoc power analysis was conducted using G*Power software (version 3.9.1.6) to assess the statistical power.

## 4 Results

The ICCs for interobserver agreement were moderate across all measurements: 0.677 for ΔADC in the supine position, 0.541 for ΔADC in the sitting position, 0.605 for ADC_mean_ in the supine position, and 0.610 for ADC_mean_ in the sitting position. Thus, the average values for ΔADC and ADC_mean_ from the ROIs defined by the two observers were used for statistical analysis. To further address the potential concern that averaging measurements might mask observer‐specific discrepancies, we performed additional analyses using each observer's measurements independently. The results showed that the positional changes in ΔADC and ADC_mean_ were statistically significant and consistent in direction for both observers (See Table S1 in the Supporting Information for individual results for each observer).

**FIGURE 4:**
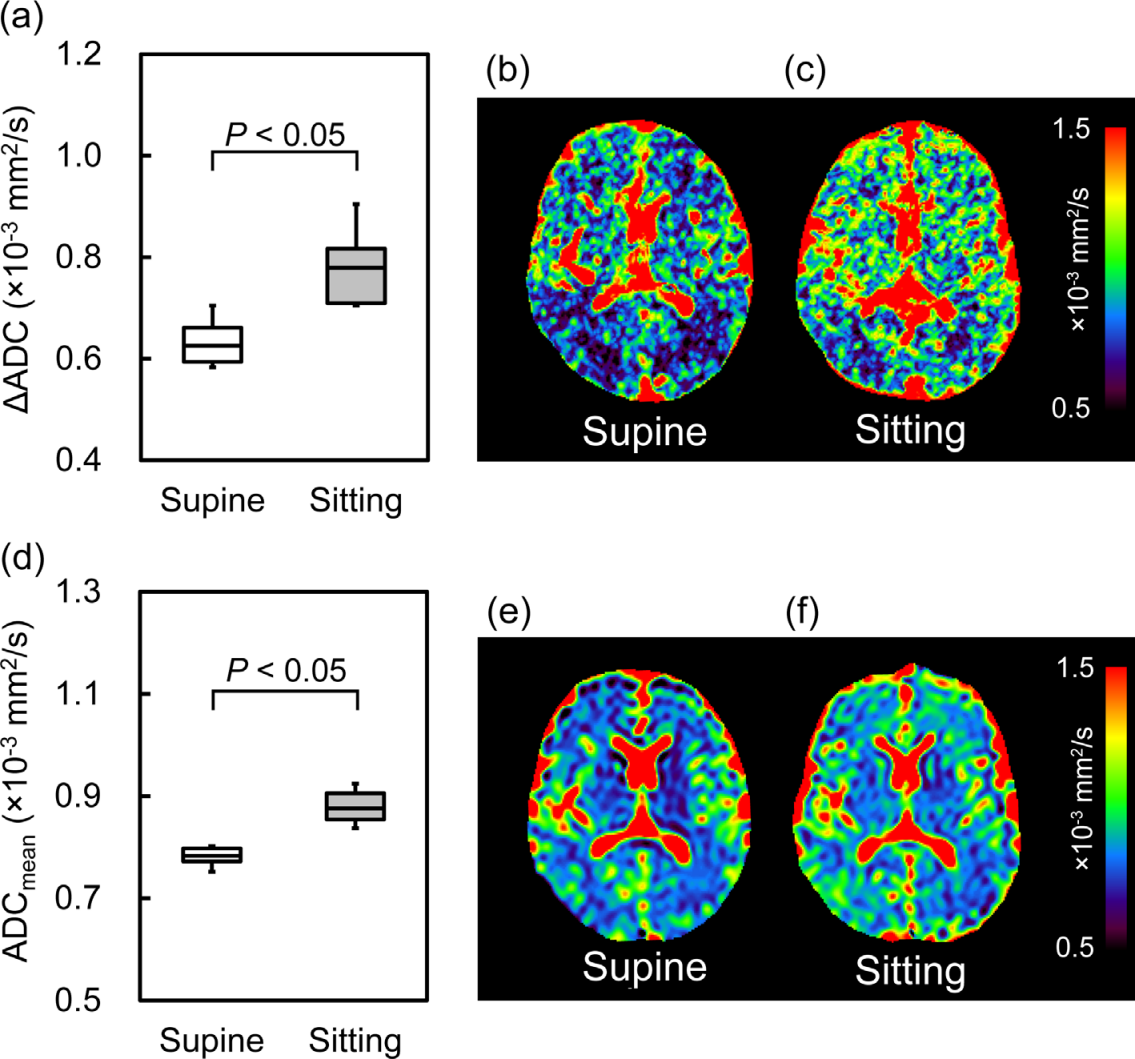
ΔADC and ADC_mean_ of the brain in supine and sitting positions. (a) Box plots of ΔADC, with representative maps in (b) supine and (c) sitting positions. (d) Box plots of ADC_mean_, with representative maps in (e) supine and (f) sitting positions.

Figure 4 illustrates the ΔADC and ADC_mean_ values and representative maps in the supine and sitting positions. There was a significant increase (24.7% ± 11.9%) in ΔADC values when participants transitioned from the supine to the sitting position (0.84 ± 0.06 × 10^−3^ mm^2^/s vs. 0.68 ± 0.05 × 10^−3^ mm^2^/s, respectively). Similarly, the ADC_mean_ was significantly increased (11.0% ± 6.3%) in the sitting position compared with the supine position (0.87 ± 0.02 × 10^−3^ mm^2^/s vs. 0.79 ± 0.06 × 10^−3^ mm^2^/s, respectively). The statistical power for ΔADC and ADC_mean_ comparisons was 0.99, and increases were consistent across all participants.

In addition, HR was significantly higher (11.0% ± 6.3%) in the sitting position than in the supine position (73.8 ± 8.4 bpm vs. 58.1 ± 3.7 bpm, respectively; see Figure S1 in the Supporting Information), with a statistical power of 0.99.

The mean SNR for the *b* = 0 s/mm^2^ images was 20.6 ± 1.9 in the supine position and 19.5 ± 1.7 in the sitting position, and for the *b* = 500 s/mm^2^ images, it was 24.2 ± 1.6 in the supine position and 22.8 ± 1.7 in the sitting position.

## 5 Discussion

This study demonstrated that the body's position affects water molecule fluctuation and diffusion in the brain. Specifically, changing from the supine to the sitting position significantly increased ΔADC and ADC_mean_ of the brain, indicating that water diffusion properties in the brain are sensitive to changes in gravitational force caused by postural changes.

Previous studies have shown that postural changes affect intracranial conditions [20, 21, 22, 24]. Alperin et al. reported that changing from the supine to the sitting position significantly increased both the ICVC and intracranial compliance (the ratio of volume to pressure changes in the cardiac cycle) [21]. Additionally, the pulsatility of venous and CSF flow notably decreased. These results suggest that the higher compliance in the sitting position allows the brain to better absorb the larger transient volume loading from arterial blood flowing into the cranium during systole, despite less pulsatile venous and CSF flow. These findings may explain the increased ΔADC of the brain in the sitting position as follows: The higher intracranial compliance in the sitting position increases the capacity to absorb volume loading, thereby allowing for a larger ICVC. Since the ICVC is the driving force for ADC changes over the cardiac cycle [25], a larger ICVC in the sitting position causes water molecules in the brain to fluctuate more, thereby increasing ΔADC. The increase in ADC_mean_ in the sitting position may also be explained by the increased fluctuation of water molecules, leading to increased ADC of the brain during the cardiac cycle. Moreover, when changing from the supine to the sitting position, the percentage increase in ADC_mean_ in the current study was smaller than the percentage increase in ΔADC. This difference is likely due to the averaging of ADC changes over the cardiac cycle in the ADC_mean_ calculation and suggests the higher sensitivity of ΔADC to postural changes. However, it should be noted that ΔADC, based on the maximum and minimum ADC values for each voxel, might be more susceptible to noise than ADC_mean_, and this difference in noise sensitivity may have contributed to the observed difference in the percentage increase between ΔADC and ADC_mean_.

ΔADC has been shown to increase even with low intracranial compliance in patients with iNPH [16]. In this pathological biomechanical property of the brain, due to the low capacity to absorb volume loading, even small volume loading causes a large pulsation force propagated directly into the brain parenchyma, resulting in greater water molecule fluctuations in the brain. Thus, while ΔADC increases in both the sitting position and iNPH, the underlying mechanism differs. Specifically, in the sitting position of healthy participants, water molecule fluctuation (output) increases because of increased volume loading (input). In iNPH, it may increase because of pathological changes in biomechanical properties (transfer characteristics between the input and output). However, to verify this hypothesis, the relationship between ΔADC, ICVC, and intracranial compliance changes in both positions should be assessed.

HR changes associated with blood flow autoregulation in the body when changing the body position could also contribute to ΔADC changes. In the sitting position, gravity redistributes blood to the lower body, reducing the venous return to the heart and blood flow to the head. The autonomic nervous system increases the sympathetic activity and HR to maintain the cardiac output and CBF [26]. This was observed in the current study where HR was increased in the sitting position compared with the supine position. Our previous study showed that the amplitude of CSF pulsation did not change between supine and standing positions [27]. Assuming this constant pulsatile amplitude, the higher HR in the sitting position implies a greater force fluctuating water molecules, potentially contributing to the increased ΔADC. Further research should explore the interplay between HR and brain water fluctuation.

This study showed larger ΔADC values (0.84 ± 0.06 × 10^−3^ mm^2^/s in the sitting position and 0.68 ± 0.05 × 10^−3^ mm^2^/s in the supine position) compared to those (ranging from 1.1 ± 0.9 × 10^−5^ mm^2^/s to 3.2 ± 1.0 × 10^−5^ mm^2^/s) reported by Sloots et al. [18], with approximately two orders of magnitude difference. This difference can be primarily attributed to two methodological factors. First, in the current study, ΔADC was calculated on a voxel‐by‐voxel basis, whereas Sloots et al. used an ROI‐based approach. The voxel‐by‐voxel approach maintains the independence of ADC waveforms between voxels, potentially providing higher sensitivity to pathological and postural physiological changes. Adjacent voxels in brain tissue do not necessarily exhibit similar ADC waveforms over the cardiac cycle due to localized physiological variations. Thus, an ROI‐based analysis averages the ADC values over a larger area, smoothing out these variations and resulting in lower ΔADC values. While Sloots et al. noted that voxel‐by‐voxel calculations of ΔADC are sensitive to noise, this study confirmed that the SNR of DWI exceeded 5.12 in both positions, which is required for reliable ADC measurements [28]. Moreover, the similar SNR between both positions suggests that noise effects and motion artifacts had a minimal contribution to the observed postural differences. Therefore, the observed changes in ΔADC between supine and sitting positions likely reflect physiological changes in intracranial conditions rather than noise effects or motion artifacts. Second, the difference in b‐values used for ADC calculations in the two studies likely contributes to the discrepancy in ΔADC. A previous study has shown that ΔADC tends to increase with lower b‐values due to increased perfusion sensitivity [29]. The current study used b‐values of 0 and 500 s/mm^2^, whereas other studies have typically used 0 and 1000 s/mm^2^ [8, 16, 17, 25], and Sloots et al. used 300 and 1000 s/mm^2^. In the current study, the use of a lower maximum b‐value (500 s/mm^2^) was necessary due to the lower static magnetic field strength of our MRI scanner (0.4 T) compared to those used in clinical settings (1.5 or 3.0 T). At 0.4 T, using a b‐value of 1000 s/mm^2^ would result in insufficient SNR, potentially compromising the accuracy of ADC measurements. Therefore, a b‐value of 500 s/mm^2^ was selected as a compromise between adequate diffusion weighting and maintaining sufficient SNR for reliable ADC quantification. Consequently, the ΔADC of the brain in the supine position (0.68 × 10^−3^ mm^2^/s) in the current study was approximately three times larger than previously reported values (0.24 × 10^−3^ mm^2^/s) obtained using a voxel‐by‐voxel analysis with b‐values of 0 and 1000 s/mm^2^ [16]. Furthermore, the inclusion of *b* = 0 s/mm^2^ likely introduced additional sensitivity to perfusion effects, potentially contributing to the larger ΔADC values. The increased sensitivity of ΔADC to perfusion at lower b‐values may suggest that the ΔADC measurements in the current study might be affected by both water molecule fluctuation and perfusion. This increased perfusion sensitivity raises the question of whether postural changes in brain perfusion may potentially affect the ΔADC measurements. However, this appears unlikely for the following reasons: Alperin et al. reported that CBF slightly decreased in the sitting position compared to the supine position [21]. In addition to this finding, another research group has demonstrated that CBF velocity, measured using Transcranial Doppler ultrasound, is also reduced in the sitting position compared to the supine position [30]. If perfusion changes were the dominant factor influencing the current results, the decreased CBF in the sitting position should result in a decrease in ΔADC. Contrary to this expectation, an increase in ΔADC in the sitting position was observed in this study. This suggests that the contribution of posture‐related changes in brain perfusion to the ΔADC measurements is small, and that other physiological factors associated with posture are responsible for the increased ΔADC.

This study indicates that evaluating ΔADC changes with body position could provide new insights into the brain's capacity to compensate for gravitational changes, that is, brain homeostasis. While MRI examinations are typically performed in the supine position, understanding the effects of body position on ΔADC and ADC_mean_ of the brain has significant implications for understanding how postural changes influence the biomechanical properties of the brain. A previous study has found that ICP changes between different body positions were greater in patients with iNPH and idiopathic intracranial hypertension than in healthy participants [24]. Similarly, postural changes in ΔADC may also vary with pathological conditions. In other words, assessing postural ΔADC changes has the potential to improve the understanding and diagnosis of disorders affecting the hydrodynamic and biomechanical properties of the brain, including posture‐dependent diseases like spontaneous intracranial hypotension [31]. In addition, the results of the current study have important implications for clinical MRI practice. While a dedicated multi‐posture MRI system was used, recent clinical MRI scanners equipped with tilt‐capable head coils allow for some degree of head‐up position for patient comfort during examination. The results of the current study suggest that such postural changes might affect ΔADC and ADC measurements through alterations in ICP. Therefore, careful consideration should be given to head position when interpreting diffusion parameters in clinical settings, particularly in cases where the head‐up position is necessary or when comparing sequential examinations where head position might have varied.

### 5.1 Limitations

First, only one combination of b‐values (0 and 500 s/mm^2^) was used for measurements. While using higher b‐values, such as the combination of 300 and 1000 s/mm^2^ employed by Sloots et al. [18], might help eliminate perfusion effects, it could also potentially reduce the sensitivity to water molecule fluctuations. Although the results of the current study suggest that perfusion effects are not the primary driver of the observed postural changes in ΔADC, future studies should focus on determining the optimal b‐value combination that can minimize perfusion effects while maintaining sensitivity to water molecule fluctuations. Furthermore, ADC might be affected by changes in T_2_ relaxation time, as reported in a previous study [32]. While postural changes in brain T_2_ could potentially influence ΔADC and ADC_mean_ measurements, quantitative T_2_ measurements were not performed in this study. Additional research incorporating T_2_ measurements in both positions would be necessary to evaluate whether postural changes in T_2_ contribute to the observed differences in ΔADC and ADC_mean_.

Second, the study was performed on a small group of young healthy males to minimize age and gender‐related variability. While this allowed for more controlled observations, it limits the generalizability of the findings. Future research should include a diverse population, encompassing various ages, both genders, and patients with brain hydrodynamic and biomechanical impairments, to validate and extend the findings.

Third, technical constraints of the DWI protocol, including single‐slice acquisition, limited spatial resolution, and image distortion, restricted the ROI placement. Specifically, due to the single‐slice acquisition, our analysis was limited to the frontal and occipital white matter at the basal ganglia level; it was not possible to evaluate ΔADC and ADC_mean_ in other regions, such as the temporal white and gray matter, due to potential partial volume effects. Advancements in MRI sequences, for example, multislice excitation techniques, higher spatial resolution, and distortion correction algorithms, would facilitate more comprehensive analyses across various brain regions in future studies, allowing for the investigation of potential regional differences in response to postural changes.

Fourth, the MRI scans were performed in a fixed order, potentially introducing order effects. While we believe the 10‐min interval between scans allowed for sufficient physiological adaptation and minimized short‐term order effects, based on previous studies showing stabilization of physiological parameters (including ICP, HR, and systemic blood pressure) within a few minutes after a posture change [33, 34], longer‐term effects cannot be completely ruled out. Therefore, future studies should consider a randomized order of body positions to address this limitation.

## 6 Conclusion

ΔADC of the brain was significantly higher in the sitting position than in the supine position.

## Supporting information


Data S1.
