# Supplementary material for: Effect of Body Position on Dynamic Apparent Diffusion Coefficient Changes During the Cardiac Cycle in the Human Brain
Source: J Magn Reson Imaging. 2025 Mar 21;62(1):295–302. doi: 10.1002/jmri.29758 (PMC12179367; doi:10.1002/jmri.29758)

| Table S1 ΔADC and ADC_mean_ values for each observer in supine and sitting positions.  *P* values were calculated using the Wilcoxon signed-rank test to compare values between supine and sitting positions for each observer. | | |
| --- | --- | --- |
|  | Observer 1 | Observer 2 |
| ΔADC (× 10^-3^ mm^2^/s) |  |  |
| Supine | 0.69 ± 0.05 | 0.66 ± 0.05 |
| Sitting | 0.86 ± 0.07 | 0.82 ± 0.05 |
| *P* value | < 0.05 | < 0.05 |
| ADC_mean_ (× 10^-3^ mm^2^/s) |  |  |
| Supine | 0.80 ± 0.08 | 0.79 ± 0.03 |
| Sitting | 0.88 ± 0.02 | 0.87 ± 0.03 |
| *P* value | < 0.05 | < 0.05 |
| ΔADC: dynamic changes in apparent diffusion coefficient (ADC) during the cardiac cycle ADC_mean_: mean ADC across all cardiac phases | | |

**Figure S1**. Heart rate (HR) in supine and sitting positions.


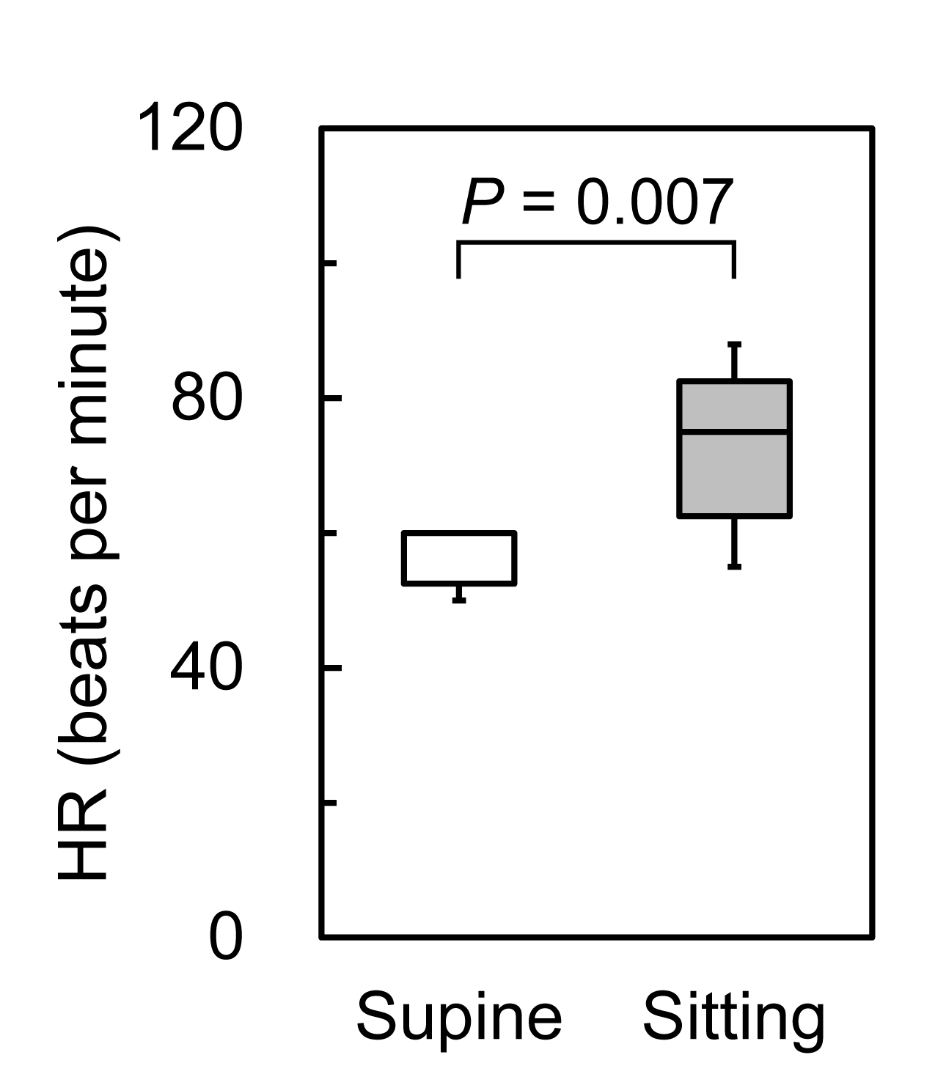

Supplement: Supplementary file 1 — Data S1. [file JMRI-62-295-s001.docx]
